# Analyzing spatial and space-time clustering of facility-based deliveries in Bangladesh

**DOI:** 10.1186/s41182-019-0170-9

**Published:** 2019-07-16

**Authors:** Atique Iqbal Chowdhury, Abu Yousuf Md Abdullah, Rafiqul Haider, Asraful Alam, Sk Masum Billah, Sanwarul Bari, Qazi Sadeq-ur Rahman, Warren Christopher Jochem, Ashraf Dewan, Shams El Arifeen

**Affiliations:** 1 Maternal and Child Health Division, International Centre for Diarrhoeal Disease Research, Bangladesh (icddr,b), Dhaka, Bangladesh; 2 School of Public Health and Health Systems, Faculty of Applied Health Sciences, University of Waterloo, Waterloo, Canada; 3 Bureau of Meteorology, Collins St, Docklands, Australia; 4 School of Geography & Environmental Science, University of Southampton, University Road, Southampton, UK; 5 School of Earth and Planetary Sciences, Faculty of Science and Engineering, Curtin University, Bentley, Australia

**Keywords:** Space-time scanning, Hot spots, Cold spots, Facility delivery, Heterogeneity, Cluster

## Abstract

**Background:**

A spatial and temporal study of the distribution of facility-based deliveries can identify areas of low and high facility usage and help devise more targeted interventions to improve delivery outcomes. Developing countries like Bangladesh face considerable challenges in reducing the maternal mortality ratio to the targets set by the Sustainable Development Goals. Recent studies have already identified that the progress of reducing maternal mortality has stalled. Giving birth in a health facility is one way to reduce maternal mortality.

**Methods:**

Facility delivery data from a demographic surveillance site was analyzed at both village and *Bari* (comprising several households with same paternal origins) level to understand spatial and temporal heterogeneity. Global spatial autocorrelation was detected using Moran’s *I* index while local spatial clusters were detected using the local Getis *G*_i_* statistics. In addition, space-time scanning using a discrete Poisson approach facilitated the identification of space-time clusters. The likelihood of delivering at a facility when located inside a cluster was calculated using log-likelihood ratios.

**Results:**

The three cluster detection approaches detected significant spatial and temporal heterogeneity in the distribution of facility deliveries in the study area. The hot and cold spots indicated contiguous and relocation type diffusion and increased in number over the years. Space-time scanning revealed that when a parturient woman is located in a *Bari* inside the cluster, the likelihood of delivering at a health facility increases by twenty-seven times.

**Conclusions:**

Spatiotemporal studies to understand delivery patterns are quite rare. However, in resource constraint countries like Bangladesh, detecting hot and cold spot areas can aid in the detection of diffusion centers, which can be targeted to expand regions with high facility deliveries. Places and periods with reduced health facility usages can be identified using various cluster detection techniques, to assess the barriers and facilitators in promoting health facility deliveries.

**Electronic supplementary material:**

The online version of this article (10.1186/s41182-019-0170-9) contains supplementary material, which is available to authorized users.

## Introduction

Bangladesh has shown remarkable success in achieving the Millennium Development Goal and reducing the maternal mortality ratio (MMR) [1]. Although, MMR in Bangladesh was sharply reduced from 322 to 194 deaths per 100,000 live births during the years 1998–2010, a recent survey conducted by the National Institute of Population Research and Training (NIPORT) found a stalled reduction for the years 2010–2016 [2]. In such a reality, achieving the target to reduce MMR to 105 in the fourth Health, Population and Nutrition Sector Program (HPNSP) by 2022 and achieving the Sustainable Development Goal (SDG) of reducing MMR to 70 by 2030 will prove to be extremely challenging for Bangladesh [3]. However, effective care during pregnancy, ensuring deliveries at health facilities, and maintaining a good quality of care in hospitals can substantially reduce maternal deaths [4] and help overcome this static condition.

In a systematic review of assessing facilitators and barriers to facility-based delivery in low- and middle-income countries, Bohren et al. [5] found women’s attitude to facility birth as a crucial factor to promote facility deliveries [5, 6]. Similarly, several studies suggested that many women personally preferred home deliveries due to three main reasons: first, because they can retain more control in the birth process; second, to avoid vaginal examinations, episiotomy, and labor in public wards that they deem as dehumanizing and violations of privacy; and third, because of the previous birth experiences, which lead them to the belief that chances of complications decline with higher birth order, and therefore, delivery at a facility after first birth would be an unnecessary luxury [5–10]. Elderly women can also discourage young parturient women from availing facility care and force them to choose home delivery for maintaining intergenerational traditions [8, 10, 11]. The influence of elderly or once-delivered women on a new to-be mother is so prominent that in many cases it overrules the husband’s decision to deliver at a health facility [8, 12–14]. Conversely, women having good experiences of delivering at health facilities were also observed to highly encourage parturient women to deliver at health facilities [5, 15, 16]. Therefore, areas with high facility deliveries have the potential to act as diffusion centers, from which, through experience sharing, facility deliveries can be promoted in adjacent areas having low facility delivery counts.

Understanding the spatial and temporal heterogeneity of facility delivery can greatly aid in the identification of spatiotemporal clusters, diffusion centers, and areas exhibiting positive home delivery trends [17]. For example, Bosomprah et al. evaluated clusters of non-facility deliveries in Ghana for targeted intervention [18]. Mwaliko et al. detected hotspots of facility deliveries in western Kenya, to ascertain the type of facility around which clusters are formed [17]. Ansariadi and Manderson identified clusters to understand the relationship between the distribution of facilities and the formation of clusters [19]. Although these studies provide insights to the spatial heterogeneity pertinent to facility deliveries, there is no clear indication as to how these clustering patterns change over time. Spatial and temporal distribution of facility delivery is important for policy makers for two critical reasons. First, it helps identify temporal patterns, which allows a detailed study of the factors influencing these patterns and assists in the identification of areas that are showing stunted progress (in terms of attracting women to deliver at facilities). For example, the proportion of facility delivery in Bangladesh was 29% in 2011 and 37% in 2014; these raw figures conform to an increasing trend [20]. However, Rahman et al. [21] studied the trends and progresses in the coverage of indicators of Universal Health Coverage in Bangladesh and strongly recommended an immediate upscaling of existing health reform initiatives. Their study found that factors such as the essential health service coverage and protection from impoverishing health service expenditures need to be prioritized, to achieve the 2030 SDG targets of reducing MMR [21]. Second, any health system reforms or upscaling initiatives in Bangladesh must address both the spatial and temporal patterns of facility delivery because previous studies have found profound temporal variations in regional inequities of maternal health care services at both the micro- and macro-scale [20, 22]. Therefore, for a resource-constraint country like Bangladesh, studying the spatial and temporal patterns of facility delivery can help policy makers identify areas with health service gaps, devise targeted interventions, and ensure proper allocation of finite and valuable resources.

Despite the wide use of spatiotemporal studies in elucidating the epidemiology of infectious diseases [23–27], there is a severe paucity of such studies, in the contextual planning, to ensure better maternal and child health. Therefore, to address the present research gap, this study aimed at answering two specific research questions. First, is there a heterogeneity or a clustering pattern in the spatiotemporal distribution of facility delivery in Bangladesh? If yes, does this multi-temporal pattern conform to a static or a dynamic nature? Therefore, the main objective of this study is to demonstrate a new approach of analyzing the distribution of facility delivery, through an application of spatial and space-time cluster detection techniques.

## Material and methods

### Study area

The study area is Mirzapur *Upazila* (analogous to sub-district) in Tangail district. The area lies in a flat floodplain and is crossed by numerous rivers. In 2018, the total population was 423,708 with a population density of 1132.9 people per square kilometer [28]. Male and female are at nearly equal proportions, having a literacy of 59.0% and 52.2% respectively, and relying heavily on agricultural activities as the main mode of livelihood (52%).

**Fig. 1:**
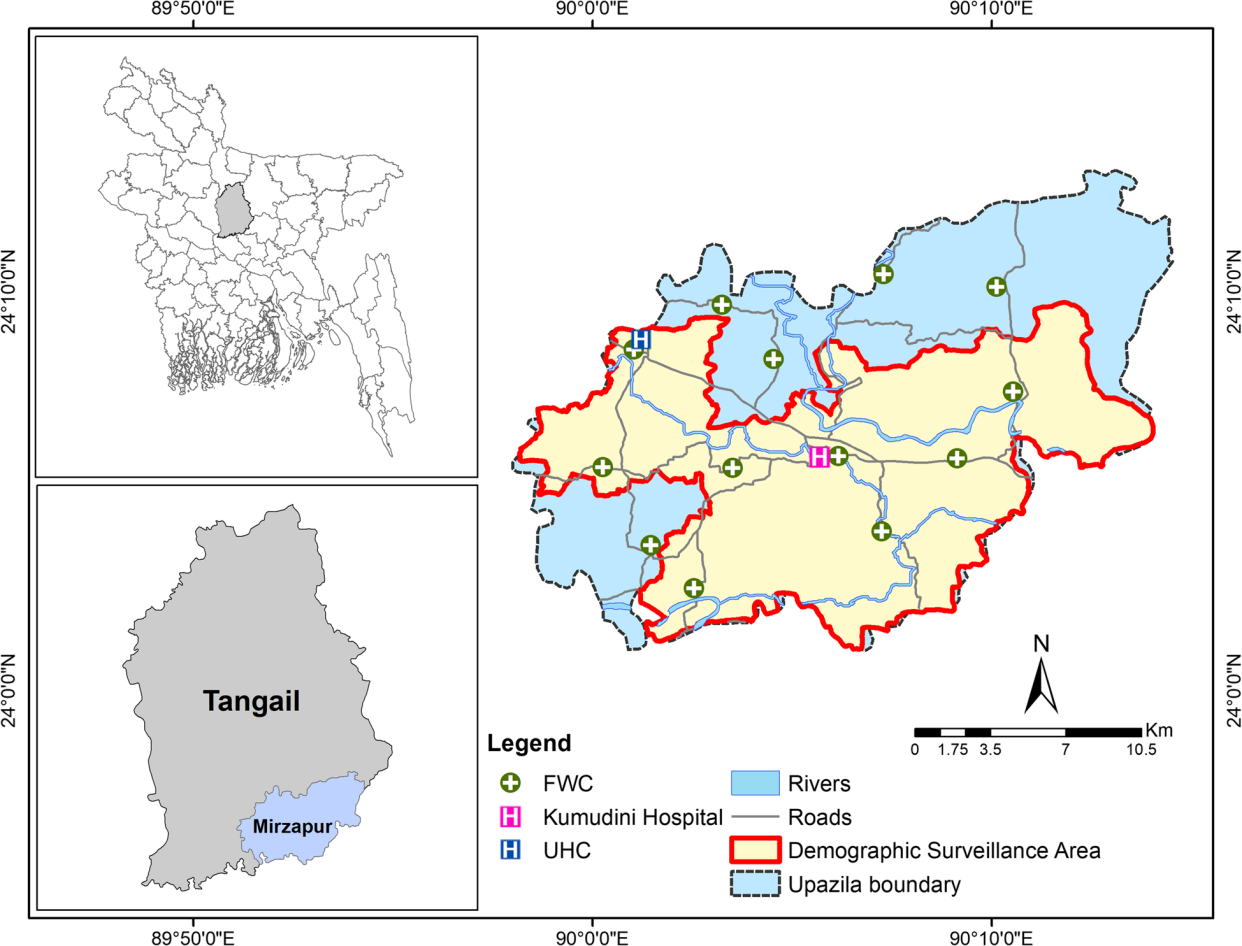
The study area map with prominent health facilities

About 52% of the deliveries take place at home and 44% take place in a health facility, the rest 4% deliver at different places such as the paternal place of the women outside the study region [29]. The majority of facility deliveries take place at the Upazila Health Complex (UHC), Family Welfare Clinic (FWC), and Kumudini (a non-profit, private hospital) [29]. Family welfare center is the first level of health facility in Bangladesh and is located at the *Union* (local administrative unit smaller than a sub-district but larger than a village) level, which is considered the lowest administrative unit in Bangladesh. Each FWC has a sub-assistant community medical officer (SACMO), paramedic, and family welfare visitor. In contrast, a UHC is a primary-level hospital facility in Bangladesh and is generally located at the *Upazila Sadar* (headquarter). UHCs provide inpatient and outpatient services, which range from 31- to 50-bed facilities depending on the population size of the *upazila*. Each UHC has several medical officers, nurses, paramedics, and administrative persons. Figure 1 shows that the health facilities in the region are evenly scattered and are well connected by all types of road networks. Although the government-subsidized UHC is located at the extreme northwestern boundary, the privately owned Kumudini hospital is situated at the central portion of the studied *upazila*.

This study employs data retrieved from a demographic surveillance system (DSS) that was established in Mirzapur since January 2007 and was implemented by the Centre for Child and Adolescent Health (presently, Maternal and Child Health Division) of the International Centre for Diarrhoeal Disease Research, Bangladesh (icddr,b). The DSS is a part of a multi-country study that aims at evaluating diarrheal disease in infants and young children in developing countries. The DSS population covers 8 out of 13 unions and is a representative of the population of the *upazila* [29].

### Study design

#### Surveillance data

We collected both the spatial and non-spatial data from the demographic surveillance system for the years 2007–2014, during the period when the surveillance was conducted by icddr,b. The surveillance system contains the geolocation of every *Bari*, which is a group of households bearing common ancestors of paternal origin. The Bari is the common organizational unit for rural Bangladesh and has been previously used to create geographic information systems [30]. As the main objective of the study is to demonstrate the dynamic nature of the facility delivery and because there is a severe paucity of studies that analyze how the clustering pattern changes simultaneously across space and time, we believe the use of an old yet very fine-scaled dataset serves our study purpose. Furthermore, the availability of such high-resolution dataset that records demographic information at an individual, Bari, and household level is quite rare for conducting a study like ours, especially in the context of rural settings in developing countries.

We extracted the DSS data relating to population and facility deliveries only and aggregated the total population and delivery data at two specific levels. First, we calculated the total population (male and female) and deliveries for each village in a single year and then joined them against a village polygon shapefile. The male and female combined population was used to analyze spatiotemporal trends in order to adjust for the population distribution within the study area, and also to understand clustering pattern of deliveries as a factor of the total population. Second, we calculated the total population and deliveries in each Bari and joined them with the Bari shapefile, produced as a point feature from the surveillance data. Additionally, we have calculated the shortest Euclidean distance between the Baris and the nearest health facility from these Baris, using the *Near* tool in the ArcMap module developed by the Environmental Systems Research Institute.

#### Analyzing spatiotemporal heterogeneity-detecting clusters of facility delivery

Before proceeding with a detailed cluster analysis, we conducted an average nearest neighbor analysis to check whether any statistically significant spatial clustering pattern exists in the study area. Once a clustering pattern was confirmed, three distinct cluster detection methods were utilized for an in-depth study of the spatial and temporal heterogeneity of facility deliveries in the study area. Moran’s *I* was first used to check the macro- or global clustering condition in the area, and second, local *G*_i_^*^ statistics detected clusters at the micro- or local level. Third, the space-time scan statistics was applied against the Bari data using the discrete Poisson model (using individual and distinct case counts). We employed three distinct cluster detection methods because past studies [17–19] have either aggregated delivery counts to a spatial unit or have used raw counts to detect clusters. Aggregating causes loss of information, as within the spatial unit, the distribution is assumed to be homogenous [19, 31]. Similarly, using only point data can either result in overfitting due to the high concentration of observation points or result in information loss when duplicate points (observations having very close geographic locations) are deleted from the analysis [32–34]. Furthermore, obtaining consistent results from different cluster-detecting algorithms has been found to be robust and constitute to higher precisions, than using a single algorithm [27, 35].

The spatial autocorrelation and the extent of overall spatial clustering were analyzed using global Moran’s *I* statistic. The test was run individually for each annual delivery datasets from 2007 to 2014. We utilized the first-order Queen’s case contiguity rule to analyze the spatial adjacency relationship and to compensate for the irregular size and shape of the villages. The global tools test the existence of overall clustering (positive or negative autocorrelation) and whether objects with similar attribute values lie close to each other [36]. Moran’s *I* range from + 1 (highly positive autocorrelation) to − 1 (highly negative autocorrelation), while a value of 0 corresponds to spatial randomness in distribution [37].

The next step involved using local *G*_i_^*^ statistic to find the villages bearing statistically significant spatial clusters [38]. The local *G*_i_^*^ statistic calculated the Getis-Ord *G*_i_* statistic and produced a *z-*score and *p* value for each village [39]. Local *G*_i_^*^ statistic compared the local mean delivery rate with the global mean rate by looking at each village within the context of the neighboring features [40]. A village produced a statistically significant and high positive *z-*score (hotspots) when it has a high count of facility deliveries and is surrounded by other features with high values as well. When a village produced a statistically significant negative *z-*score, it contained clusters of low values surrounded by other villages with low values of delivery counts (cold spots) [41, 42].

Finally, the space-time cluster analysis was carried out using the spatial scan statistic implemented in SaTScan (version 9.4) [43, 44]. The probability model was set to discrete Poisson and was set to detect the clusters with high rates (for this study, the clusters with high facility deliveries). This method has increasingly been used by epidemiologists and others to detect regions with significantly elevated disease rates [27, 45]. However, its use in detecting space-time clusters of facility delivery is almost non-existent. SaTScan detected space-time clusters using cylindrical scan windows with a circular geographic base and the height corresponding to some interval in time [46]. In these scans, geographical locations of the Baris were considered as points bearing the number of cases (facility delivery case) and the population at various times. The circular scan window (base of the cylinder) moved throughout the space while varying the cylinder’s radius and time duration. The likelihood ratio was calculated with the null hypothesis that the rate of facility delivery is the same inside and outside the scan window [45]. The window producing the maximum likelihood was identified as the most significant cluster and was known as the primary cluster, while the other significant clusters with lower maximum likelihood than the primary was reported as the secondary clusters [43, 44]. The *p* values of these clusters are produced by Monte Carlo replications of the datasets to measure the statistical significance of the clusters [47]. In this study, we set the maximum cluster size as 50% of the population at risk. For delivery data, this would imply that the base of the scan window would increase itself to incorporate at maximum 50% of the population. We employed time precision of 1 day for reading each case dates and a time aggregation of 6 months to fix the temporal window to 1 year, for the ease of interpretation. Based on our field experiences and the findings from previous studies on poor birth preparedness in rural Bangladesh [48, 49], we hypothesized that it would take around 3 months for a woman to detect her pregnancy and another 3 months for her family to decide her delivery place. Therefore, a time aggregation of 6 months had been used. We believe that within this 6 months interval, a parturient woman is most influenced by another woman having delivered at a facility. The scan calculated *p* value using Monte Carlo replication of 999 times and was restricted to avoid any geographical overlapping of the detected clusters. Adjustments for confounders were made by adjusting for the birth parity and educational and economic conditions of the households. Education was measured as the duration of the study (in years) of the household head, and the economic condition was derived from asset scores of the households (see Additional file 1 for details). The education status of the household head was used for the adjustment because in rural Bangladesh, the household head mostly takes the final decision as to whether the parturient women would deliver in a health facility or home.

A sensitivity and specificity analysis was conducted to validate the space-time clustering model obtained from SatScan analysis. The methodology used for this purpose was proposed by Chen et al. [50] and can be used to evaluate space-time permutation models involving consecutive time intervals. As we had employed a time precision of 1 day in our space-time model, with a time aggregation of 6 months to achieve a temporal window of 1 year, our model deals with similar successive or consecutive time intervals required for the analysis. In order to calculate the sensitivity and specificity of the model, the following steps were followed:

- All the villages that had exhibited spatial heterogeneity in local clustering analysis (using local *G*_i_^*^ statistic) were identified. Therefore, if a village was either a hotspot or a cold spot in any of the study years, it was considered as a village with a cluster of facility delivery. The results for spatial heterogeneity derived from local *G*_i_^*^ analysis, of the individual years, were combined to obtain the spatiotemporal clustering pattern. This was finally compared with the space-time model developed from SatScan analysis. As SatScan analysis is predominantly used for the identification of space-time clusters in infectious diseases [23–27] and its use to study the distribution of facility delivery is rare, we have taken the results from local *G*_i_^*^ analysis to evaluate our space-time model.
- The villages that were identified as cluster zones using both SatScan and local *G*_i_^*^ analysis were considered as the true positives (TP). In contrast, the villages that were not identified by any of these two cluster detection techniques were considered to be true negatives (TN). False positives (FP) and false negatives (FN) were calculated from TP, TN, and the total number of villages, which were separately identified by these two techniques (see Additional file 1 for details).

## Results

After validation and verification checks, the DSS data comprised of a total of 41,600 delivery cases and 18,003 observations for individual Baris. Amongst these Baris, 8660 Baris had at least one facility delivery during the study period and were used for the cluster analysis. The number of Baris, with a facility delivery, showed a very small increase (by a rate of below 5% per year) during the study years. The locations of all deliveries are shown in Table 1.

**Table 1:** Location of all deliveries (2007–2014) in Mirzapur DSS area

Serial No.	Number of deliveries	Percentage	Location of the delivery
1	12782	30.7	Own house inside Mirzapur
2	7643	18.4	Kumudini hospital
3	4903	11.8	Any hospital/clinic outside Mirzapur
4	4847	11.7	Parents home inside Mirzapur
5	4360	10.5	Private clinic inside Mirzapur
6	3766	9.1	Parents home outside Mirzapur
7	1554	3.7	Upazila Health Complex Mirzapur
8	1513	3.6	Location could not be determined
9	232	0.6	Home outside Mirzapur

### Spatial-temporal clustering

#### Global Moran’s *I*

**Fig. 2:**
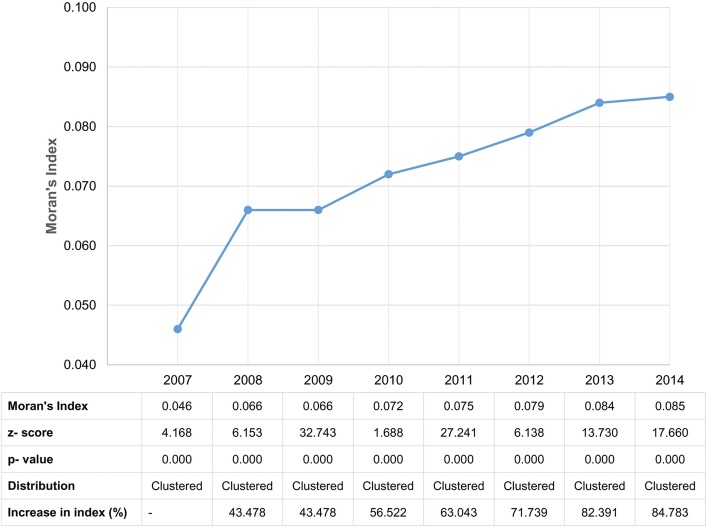
Inter-year comparison of Global Moran’s *I* autocorrelation test (with 2007 as the base year)

Figure 2 illustrates the results from Moran’s *I* spatial autocorrelation test, demonstrating statistically significant clustering during each year and a general trend toward stronger clustering.

The Moran’s *I* values showed a progressive increase throughout the study years. During the years 2007–2014, the Moran’s *I* values showed an 84.8% increase compared to the base year and a sharp increase beginning from 2009. These trends in Moran’s *I* value are an indication of persistent spatial autocorrelation in the study area.

#### Local Getis Ord *G*_i_^*^

**Fig. 3:**
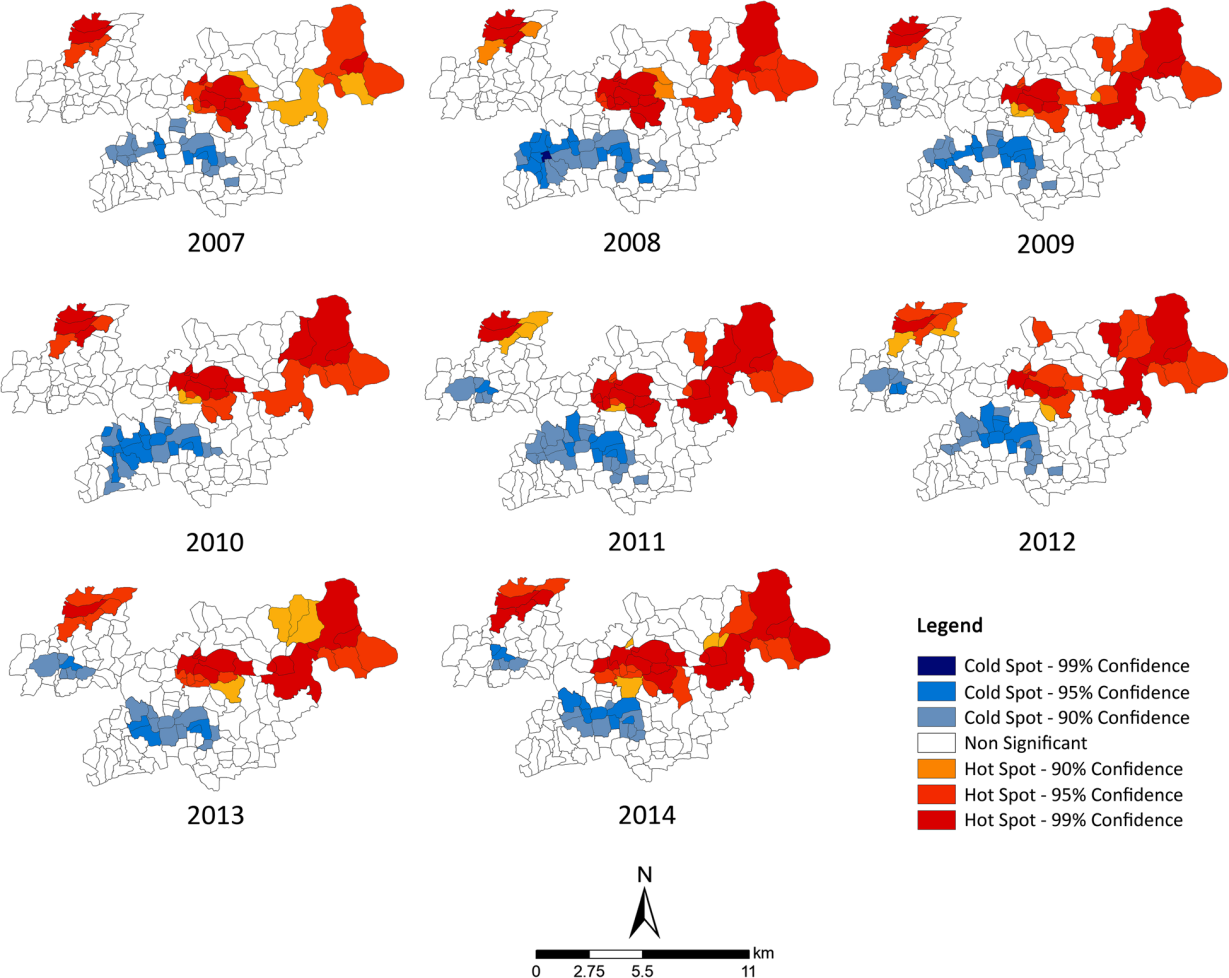
Spatial and temporal distribution of hot and cold spots in the study area

Local clustering analysis using Getis Ord *G*_i_^*^ statistics gave valuable insights into the prevailing clustering condition at the village level. The local clustering revealed facility delivery rates showing considerable heterogeneity both spatially and temporally. Figure 3 shows that despite some hot spots and cold spots appearing and disappearing in several villages over the years, there had been regions of sustained high clustering in the central, northeast, and northwestern parts of the study area. In addition, there had been regions of facility deliveries surrounded by high frequencies of home deliveries or cold spots. Table 2 summarizes temporal patterns of the number of villages with hot and cold spots areas. Although the number of cold spot villages increased from 2007 to 2008, there was no definite overall increase or decreasing trend. However, the number of villages with hot spots jumped from 2007 to 2008 but then decreased from 2008 to 2009, which was followed by an overall increasing trend for the next 5 years. Comparing each year with the base year 2007 in Table 2, with the exception of 2010, every year demonstrated an increase in the percentage of hot spot areas. The percent increase in hot spot areas at the ending year 2014 was more than three times than the beginning year of comparison, 2008. However, the cold spot areas showed an average increase of 32% from the base year until 2012, after which the increase stopped completely. As a result of this increasing trend of the hot and cold spot areas, the percent of non-significant or non-cluster areas decreased subsequently throughout the study areas.

**Table 2:** The changes in hot spots and cold spots during the study areas (2007–2014)

Year	Hot spots		Cold spots		Non-significant	
	Number of villages	Percent change*	Number of villages	Percent change*	Number of villages	Percent change*
2007	25	–	20	–	110	–
2008	28	12.0	29	45.0	98	− 10.9
2009	26	4.0	24	20.0	105	− 4.5
2010	25	0	25	25.0	105	− 4.5
2011	27	8.0	29	45.0	99	− 10.0
2012	28	12.0	25	25.0	102	− 7.3
2013	31	24.0	20	0	104	− 5.5
2014	35	40.0	20	0	100	− 9.1

*With 2007 as the base year

#### Space-time scan analysis

**Fig. 4:**
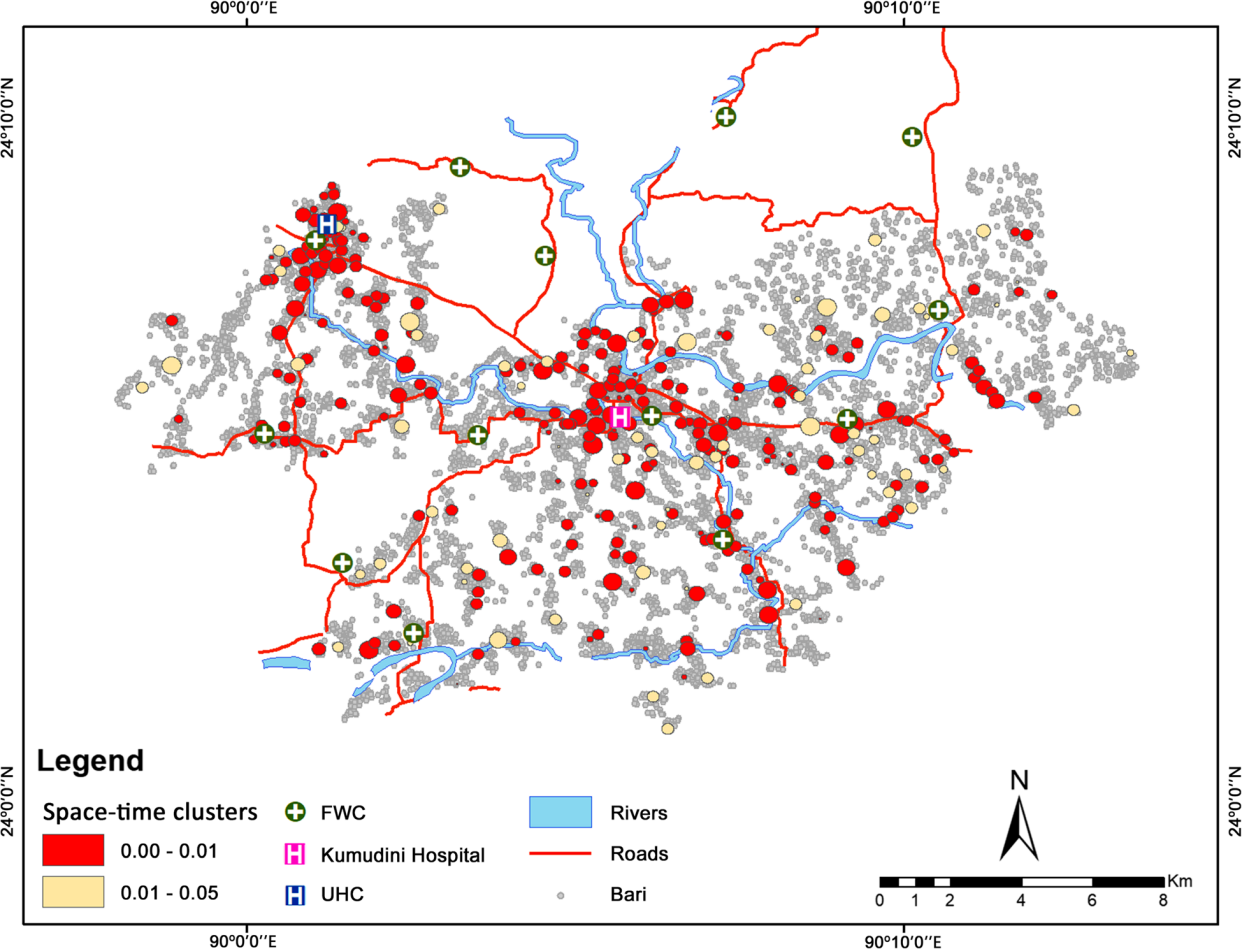
Space-time clusters of facility deliveries

The space-time scanning considered a total of 18,003 facility deliveries taking place from 8,660 Baris at different health facilities during the years 2007–2014. Amongst these, a total of 3,705 facility deliveries fell within the space-time clusters. The scan detected a total of 500 space-time clusters, amongst which 327 clusters (65.40%) were statistically significant. Figure 4 shows that the majority of the clusters (51.20%) were very highly significant (*p* < 0.01), with the clusters found all over the study area but at increasing numbers near the health facilities. The mean observed to expected ratios (ODE) of the statistically significant clusters were 27.67, and thus, the observed number of facility delivery cases within the clusters was 27.67 times higher than the expected number of cases within the scanned region.

**Fig. 5:**
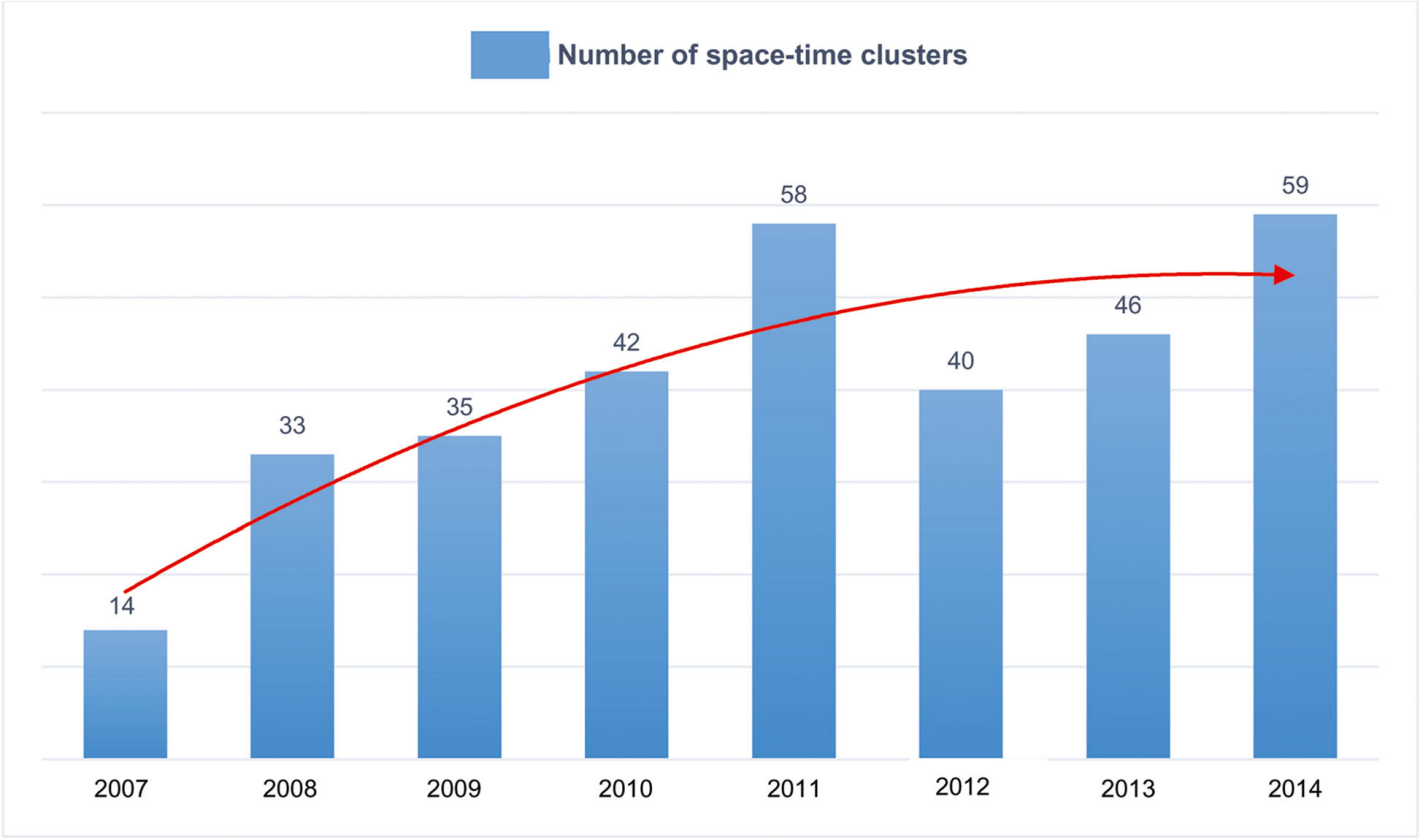
The number of space-time clusters during the study years. The red line represents the second-order polynomial curve showing an overall increase in the numbers of clusters

Figure 5 illustrates how the total number of space-time clusters varied over the individual years. The number increased sharply from 2007 to 2011, after which there had been a fall in 2012, followed by an increase in the succeeding years. The highest number of clusters detected was in the ending year 2014, which was about four times higher than that in 2007. The general trend of the annual number of clusters can be seen from the second-order polynomial curve that shows a net increase throughout the years but a decelerated cluster formation since 2011.

#### Sensitivity and specificity analysis

Out of the total 154 villages in the study area, a total of 67 villages were identified with clusters by both the SatScan and the local *G*_i_^*^ analysis (true positives). In contrast, these two analyses have identified only 14 villages, which did not exhibit any clustering pattern over the study years (true negatives). SatScan had identified 57 villages that were not present in the local *G*_i_^*^ analysis (false positives), and finally, 16 villages were not detected by SatScan that were found with clusters using local *G*_i_^*^ technique (false negative). The results are tabulated in Table 3.

**Table 3:** Results of the sensitivity and specificity analysis

		Using local *G*_i_* analysis		
		Villages with cluster	Villages without any cluster	Total
Using SatScan analysis	Villages with cluster	67	57	124
	Villages without any cluster	16	14	30
	Total	83	71	154

Using these values (Table 3), the sensitivity and specificity for the space-time model (developed from SatScan) were 80.7% and 19.7% respectively.

## Discussion

Although the application of spatial and space-time scan statistics is quite prominent for studying infectious diseases [27], their use in investigating spatial and temporal trends of facility deliveries is quite limited. This study can provide important guidelines in studying the heterogeneity of deliveries and thus undertake policy measures for targeting diffusion centers to facilitate the growth of facility delivery clusters, as well as regions of low facility deliveries that cannot be detected by conventional non-spatial techniques. We aimed at analyzing the spatiotemporal heterogeneity of facility deliveries and found that regions of a sustained hot spot or high facility delivery foci can spatially spread throughout a region over the years. In addition, our space-time cluster analysis revealed a crucial finding that clusters of facility deliveries can appear at regions distant from the health facilities. This calls for a discourse to closely examine the non-distance factors that could stall the progress of facility usage, once issues related to physical connectivity with health facilities are addressed.

The three cluster detection techniques, despite having different algorithms, established the presence of spatial heterogeneity in the study area. As suggested by Rainey et al. and Dewan et al., employing different clustering algorithms to reach the same results constitute to the robustness of our obtained results [27, 35]. The results from the sensitivity and specificity analysis show that the space-time model had a very satisfactory agreement with the models derived from the local *G*_i_^*^ analysis. A specificity of 80.7% implies that the space-time model was able to identify 80.7% of the villages that truly have a cluster [51]. The low specificity value of 19.7% reflects that the model is not well suited to identify villages that do not have any clusters, and was able to identify only 19.7% such villages [52]. This low specificity value is quite expected as the space-time model was specifically developed with a prime focus to detect only the space-time clusters in the distribution of a feature [32, 34, 43, 44].

The low Moran’s *I* values reported in the global cluster analysis could be due to the Moran’s computation mechanisms for spatial autocorrelation. Chen suggests that the results of spatial autocorrelation from Moran’s *I* can differ from the local value, due to the limitation of the method to develop a spatial contiguity matrix and to incorporate geographic scales in its function [53, 54]. Therefore, the low Moran’s index values may arise from considering the entire study area globally and being unable to incorporate the scale into the calculation. Furthermore, this finding strongly emphasizes on studying the distribution of delivery cases at a local or micro-scale, as the hot and cold spots can reduce each other’s effect and thus fail to detect any spatial heterogeneity [38, 42, 55].

In our local clustering study, using Getis *G*_i_^*^ statistics, both the hot and cold spots showed a marked contiguous diffusion pattern over the study years. The same results were found in SatScan analysis that showed an increase in the number of space-time clusters over the years. Although our study was not designed to ascertain the causes of this diffusion pattern, but given that the number of health facilities remained constant throughout the study years and the effect of both the population and distance were found to be statistically insignificant with the number of facility deliveries during our exploratory analysis, our observed diffusion patterns could be owing to the sharing of care experiences by delivered women. Interestingly, Bohren et al. [5] found a strong influence of previously delivered women on parturient women and personal links acting as a promoter of health facility usage that diffuses both across generations and geographic bounds. Our observed diffusion could also be triggered by message diffusion amongst men, whose wives had delivered at health facilities. A husband plays an important role in deciding the location of deliveries [5, 9, 15, 56], and thus, men’s positive perception and experiences of delivery services can greatly promote facility usage and encourage other prospective fathers to choose health facilities as the location of deliveries [57, 58]. Consequently, future studies focusing on the dynamics or factors affecting the spatiotemporal distribution of facility delivery clusters are expected to shed valuable insights into the actual dynamics of this observed pattern.

Furthermore, the cold spots identified in this study could be important regions of future interventions. In contrast to hot spots, cold spots are regions with features having high values that are surrounded by low values [42, 59]. Therefore, these are pockets within the villages where few facility deliveries existed in the midst of large numbers of non-facility deliveries. Despite a small relocation type diffusion exhibited by the cold spots in the southwestern part of the study area, some villages were persistent cold spots. However, it was observed that the annual percentage change in the number of cold spot villages with respect to the base year 2007 stopped at 2012 and yet, that of the hot spots continued to increase till 2014. Interventions in these areas for promoting facility deliveries could have sustained the increase of the cold spots and with a sufficient number of facility deliveries could have resulted in their conversion to hot spot areas.

Promoting facility usage by targeting diffusion centers and the application of cluster-based approaches are quite valuable in the context of countries like Bangladesh because the demographic surveillance could not be established in most of the districts and sub-districts. Additionally, any spatiotemporal study in Bangladesh to identify the service gap areas and poor facility utilization is impeded by lack of geographic data [60, 61]. In such realities, a retrospective spatial and temporal analysis conducted with the delivery data extracted from the health facilities can help identify potential areas requiring interventions. Our study identified the year from which clustering pattern in the DSS area (Moran’s *I* value) gained prominence, as well as the year from which the local level clustering (number of villages with space-time clusters) experienced a sharp fall. These marked distributional changes are hardly detected by non-spatial analysis that employs raw count data of deliveries [27, 42, 59].

Despite the numerous strengths of our study, further improvements are possible. First, the study was designed to understand the spatial and temporal distribution of facility deliveries in a rural setting of Bangladesh and had no provision to study the factors associated with the observed patterns. However, the findings and the methodology adopted in this study could still act as a basis for future studies aimed at understanding the determinants of spatial and temporal clustering of facility and home deliveries. Second, we only considered facility deliveries that took place at health facilities within the study areas and thus leading to exclusion of cases where deliveries took place at facilities outside the study areas (for example, women residing within the study area but delivering at health facilities elsewhere). As most women delivered at facilities near to their homes, we believe that the number of cases excluded was small. In addition, a large number of missing cases are required to effect the results of both the Getis *G*_i_* and SatScan algorithms [38, 42, 44]. Third, SatScan detected space-time clusters that had circular bases [34]; real-life clusters could be irregular shaped as well [62]. Despite our restricted settings of geographical overlapping of clusters in SatScan analysis, few clusters were found to overlap along their borders. This could have occurred due to the limitation of the algorithm in dealing with such high-resolution data over a large geographic area. Finally, we only checked for the influence of the total population and distance to the nearby health facility on the number of facility deliveries cases. There could be other covariates as well, but several past studies have emphasized on the distance and total population to be the major predictors of the location of deliveries and health facility usage [5, 61, 63].

In spite of the limitations, our study is one of the very few attempts to employ spatial statistics in the study of delivery cases. This study identified hot and cold spots areas and established their diffusion patterns throughout the study years, thus providing valuable information for public health officials to devise intervention strategies in targeting regions of low facility deliveries. Furthermore, we used both the village and Bari level as the geographic units of the study, and thus, our findings have wider applicability in the context of Bangladesh, where local- and micro-level socioeconomic and cultural factors can greatly influence individual choices of delivery locations. The methods adopted in this study can be generalized easily for any developing country's context and can be reproduced to understand distributions of both home and facility deliveries.

## Conclusion

The use of spatial statistics in studying the distribution of deliveries remains heavily unexplored. Studies simultaneously attempting to understand the spatial and temporal dimensions of delivery distributions are even fewer in number. For a country like Bangladesh that has shown remarkable progress in reducing maternal mortality ratio, understanding the distribution of facility deliveries can ensure the continuum of the progress and garner considerable attention towards regions with stalled facility usage. In this study, clusters were identified to establish the greater chances of delivering at a health facility, when located inside a cluster. Furthermore, years of low facility usages were identified through cluster detection processes, which could be studied further, to understand the effect of macro- and micro-scale barriers in impeding health service utilization. The findings from this study offer new insights to the way facility delivery can be analyzed in a resource-constraint country, which have a huge implication in the identification of health service gaps and to ensure proper allocation of finite resources, to ensure better maternal and child health outcomes.

## Additional file

Additional file 1: Supplementary file. (DOCX 17 kb)

## Data Availability

The datasets used and/or analyzed during the current study are available from the corresponding author on reasonable request.

## Abbreviations

MMR: Maternal mortality ratio
ODE: Observed to expected ratio
SatScan: Space-time scan
