# Supplementary material for: Analyzing spatial and space-time clustering of facility-based deliveries in Bangladesh
Source: Trop Med Health. 2019 Jul 16;47:44. doi: 10.1186/s41182-019-0170-9 (PMC6636060; doi:10.1186/s41182-019-0170-9)
Supplement: Supplementary file 1 — Supplementary file. (DOCX 17 kb) [file 41182_2019_170_MOESM1_ESM.docx]

1. **Formula used for calculating Moran’s *I*:**

| $I=\frac{n\sum_{i=1}^{n} \sum_{j=1}^{n} w_{ij}(x_{i}-\bar{x})(x_{j}-\bar{x})}{\left( \sum_{i\neq j} \sum w_{ij} \right)\left( \sum_{i=1}^{n} (x_{i}-\bar{x})^{2} \right)}$ |  |
| --- | --- |

Here, *n* is the total number of villages in the study, *i* and *j* represents different villages, *x_i_* is the facility delivery event in unit *i*, and $\bar{x}$ is the mean case incidence. *w_ij_* is a measure of the spatial proximity of pairs *i* and *j*. Thus, in global Moran’s *I*, the tendency of a village and its neighbors to differ systematically from the mean of the data set was calculated.

1. **Formula used for calculating Getis-Ord Gi* statistic:**

The Getis-Ord Gi* statistic was calculated with the following equation:

| $G_{i}^{*}=\frac{\sum_{j=1}^{n} w_{i,j}x_{j}-\bar{X}\sum_{j=1}^{n} w_{i,j}}{S\sqrt{\frac{[n\sum_{j=1}^{n} w_{i,j}^{2}(\sum_{j=1}^{n} w_{i,j})^{2}]}{n-1}}}$ |  |
| --- | --- |

Where, *x_j_* is the attribute value for village *j*, *w_i,j_* is the spatial weight between villages *i* and *j*, n is the total number of delivery cases. $\bar{X}$ is the global mean and *S*  is the standard deviation.

1. **Formula used to calculate sensitivity and specificity**

$$Sensitivity=\frac{\mathrm{TP}}{TP+FN}; Specificity=\frac{\mathrm{TN}}{TN+FP}$$

Where,

TP = True positives or the total number of villages that were identified as cluster zones using both SatScan and local *G_i_^*^* analysis.

TN = True negatives or the total number of villages that were not identified by any of the SatScan or local *G_i_^*^* analysis.

FP = False positives or the total number of villages that were identified by the SatScan analysis but not the local *G_i_^*^* analysis.

FN = False negatives or the total number of villages that were not identified by the SatScan analysis but were identified by the local *G_i_^*^* analysis.

1. **Method used to calculate the asset index of a household**

The economic status of a household was derived by calculating an asset score. Data were collected on different household conditions and the possession of various durable goods at the household level. These included:

1. Materials used for housing construction (recorded through observations by the data collector during the demographic and health survey): The materials used to build the roof, wall and the floor were categorized to natural, rudimentary and finished materials. The natural materials were considered to be comprised of either thatch, leaf, cane, palm or tree trunks. The rudimentary category contained earth, clay, mud, bamboo, jute stick, wood planks or stones. The finished materials were made up of tin, polished wood, ceramic tiles, cement, concrete or brick.
2. Access to the types of water sources and sanitation facilities (owned/shared at inside/outside the dwelling).
3. Ownership of:
4. Electrical appliances (radio, television, refrigerator, cellphones, telephones, electric fans, computers and laptops).
5. Transport vehicles (rickshaw, auto-rickshaw, van, boat, bicycle, motorcycle and electric bike).
6. Livestock (bulls, cows, goats, sheep, chickens, ducks, pigeon, and quail).
7. Homestead, agriculture and nonagricultural land.

These goods were then used to construct a proxy measure for the economic status of the household. Principal component analysis was conducted to generate a weight for each durable asset. The weights were the standardized first principal component of the variance-covariance matrix of the observed household assets. Using the weights, an asset index was created using the following formula:

*A_i_=γ_1_x_1i_+…+γ_k_ x_ki_*

Where, *A_i_* is the asset index for household *i*, the *x_ki_*'s are the *k-th* asset of that household, and the *γ*'s are the weights.

Finally, the households were ranked based on the total score of the household in which they resided and divided into wealth quintiles (ranking from lowest to highest).
